# Prospective Comparison of Thulium and Holmium Laser Lithotripsy for the Treatment of Upper Urinary Tract Lithiasis

**DOI:** 10.1016/j.euros.2023.02.012

**Published:** 2023-03-21

**Authors:** Bertrand Delbarre, Faris Baowaidan, Thibault Culty, Lotfi Khelfat, Marie Brassier, Matthieu Ferragu, Alexandre Magnier, Alexandre Secourgeon, Francois Tariel, Souhil Lebdai, Pierre Bigot

**Affiliations:** Department of Urology, Angers University Hospital, Angers, France

**Keywords:** Urolithiasis, Ureteroscopy, Laser lithotripsy, Holmium, Thulium, Stone free

## Abstract

**Background:**

Lithotripsy with holmium:yttrium-aluminum-garnet (Ho:YAG) laser is the current gold standard for treating stones of the upper urinary tract (UUT). The recently introduced thulium fiber laser (TFL) has the potential to be more efficient and as safe as Ho:YAG.

**Objective:**

To compare the performance and complications between Ho:YAG and TFL for UUT lithotripsy.

**Design, setting, and participants:**

This was a prospective single-center study of 182 patients treated between February 2021 and February 2022. In a consecutive approach, laser lithotripsy was performed via ureteroscopy with Ho:YAG for 5 mo, and then with TFL for 5 mo.

**Outcome measurements and statistical analysis:**

Our primary outcome was stone-free (SF) status at 3 mo after ureteroscopy with Ho:YAG versus TFL lithotripsy. Secondary outcomes were complication rates and results regarding the cumulative stone size. Patients were followed at 3 mo with abdominal imaging (ultrasound or computed tomography).

**Results and limitations:**

The study cohort comprised 76 patients treated with Ho:YAG laser and 100 patients treated with TFL. Cumulative stone size was significantly higher in the TFL than in the Ho:YAG group (20.4 vs 14.8 mm; *p* = 0.01). SF status was similar in both groups (68.4% vs 72%; *p* = 0.06). Complication rates were comparable. In subgroup analysis, the SF rate was significantly higher (81.6% vs 62.5%; *p* = 0.04) and the operative time was shorter for stones measuring 1–2 cm, whereas the results were similar for stones <1 cm and >2 cm. The lack of randomization and single-center design are the main limitations of the study.

**Conclusions:**

TFL and Ho:YAG lithotripsy are comparable in terms of the SF rate and safety for the treatment of UUT lithiasis. According to our study, for a cumulative stone size of 1–2 cm, TFL is more effective than Ho:YAG.

**Patient summary:**

We compared the efficiency and safety of two laser types for the treatment of stones in the upper urinary tract. We found that stone-free status at 3 months did not significantly differ between the holmium and thulium lasers.

## Take Home Message

Lithotripsy with Ho:YAG laser is the current gold standard for stones in the upper urinary tract (UUT). Thulium fiber laser (TFL) has the potential to be more efficient than and as safe as Ho:YAG. We found that TFL and Ho:YAG lithotripsy were comparable in terms of their stone-free rate and safety for treatment of UUT lithiasis. A cumulative stone size of 1–2 cm could be an argument for using TFL rather than Ho:YAG.

## 1 Introduction

For many years, ureteroscopy (URS) with holmium:yttrium-aluminum-garnet (Ho:YAG) laser lithotripsy has been considered the treatment of choice for the management of most upper urinary tract (UUT) lithiasis [1]. Thulium fiber laser (TFL) has recently been introduced for the treatment of urinary lithiasis. With US Food and Drug Administration approval in 2019 and European CE mark approval in 2020, TFL technology is now a commercially available option for stone lithotripsy. The wavelength difference between TFL (1940 nm) and Ho:YAG (2100 nm) means that TFL energy is highly absorbed by water over a shorter distance [2]. Initial in vitro studies showed that, for the same energy settings, TFL was twice as efficient for stone fragmentation and two to four times as efficient for dusting as Ho:YAG laser [3]. Four recently published clinical studies support the role of TFL as an efficient modality for lithotripsy, with no complications specific to this laser type observed in the studies [4], [5], [6], [7]. The advantages of TFL over Ho:YAG laser (ablation efficiency, less retropulsion) could lead to a reduction in operative time and to expansion of the possibilities for treating larger kidney stones with retrograde intrarenal surgery [8]. In the current study, we prospectively compare the performance of TFL in terms of the stone-free (SF) rate and safety to that of Ho:YAG laser for URS treatment of UUT lithiasis.

## 2 Patients and methods

After institutional data protection and ethics committee approval, the LiThuHol Trial (NCT04871984) prospectively included all patients treated with URS and laser lithotripsy for renal and/or ureteral stones between February 2021 and February 2022 in a single academic center. The exclusion criteria were urological anatomic abnormalities, pregnancy, age <18 yr, and an untreated positive urine culture. For the first 5 mo, we included patients who had URS with Ho:YAG laser lithotripsy (Medilas H 20; Dornier MedTech). Then we used TFL for 2 mo without including patients treated via URS (training period). Finally, in the past 5 mo, we included all patients undergoing URS and TFL (Soltive Premium 60 W; Olympus, USA) lithotripsy. The anesthesia record was used to extract preoperative data (age, sex, body mass index, American Society of Anesthesiologists score, comorbidities, and anticoagulation). Every patient underwent computed tomography (CT) before the surgery to assess and characterize their stones (localization, largest stone size, and cumulative stone size). Stone size was measured in the axial or coronal plane and the largest size was recorded. We summed the largest size for each stone to calculate the cumulative stone size. Twelve different surgeons performed the URS under general anesthesia, and patients received prophylactic antibiotics according to guideline recommendations. The surgeon was considered as a senior after 60 URS procedures [9]. Rigid URS was performed with a 7-Fr ureterorenoscope (Karl Storz). Flexible URS was performed with a digital reusable 7.5-Fr endoscope (Flex-X2; Karl Storz) or a digital single-use 9-Fr endoscope (LithoVue; Boston Scientific). A safety wire was always used, usually a 0.035-inch Terumo guidewire (Nitinol stiff guidewire; Radiofocus). Surgeons were free to use a ureteral access sheath or not in flexible URS, according to their routine practice. A dedicated form for gathering intraoperative data was completed after the surgery by the surgeon or the resident. Type of procedure, surgeon experience, operative time, laser type, disposable equipment, fiber size, intraoperative complications (bleeding, ureteral injuries, contrast extravasation), postoperative JJ stent use, and length of stay were collected. Laser settings were left to the discretion of the surgeon for Ho:YAG and TFL lithotripsy. The gravity-based irrigation method was used in each URS procedure. Ambulatory cases involved patients who were admitted and discharged on the same day. Postoperative pain was evaluated by a nurse using a verbal rating scale. Postoperative complications were recorded according to the Clavien-Dindo classification [10]. In accordance with the Lithiasis Committee of the French Association of Urology (CLAFU), all patients were seen at 3 mo for metabolic evaluation, stone composition analysis, and abdominal imaging (ultrasound or CT) [11]. SF status was defined as the absence of residual fragments or residual fragments <3 mm that were asymptomatic [12]. The primary outcome was SF status at 3 mo after URS with Ho:YAG versus TFL lithotripsy. We also performed subgroup analyses by stone size (<1 cm, 1–2 cm, and >2 cm).

Continuous variables are reported as the mean and standard deviation, and categorical variables as the frequency and proportion. Comparisons between the Ho:YAG and TFL groups were performed using the χ^2^ test or Fisher’s exact test for discrete variables, and a t test or Mann-Whitney *U* test for continuous variables, as appropriate. Analyses were performed using SPSS version 15.0 software (IBM Corporation, Armonk, NY, USA).

## 3 Results

**Fig. 1:**
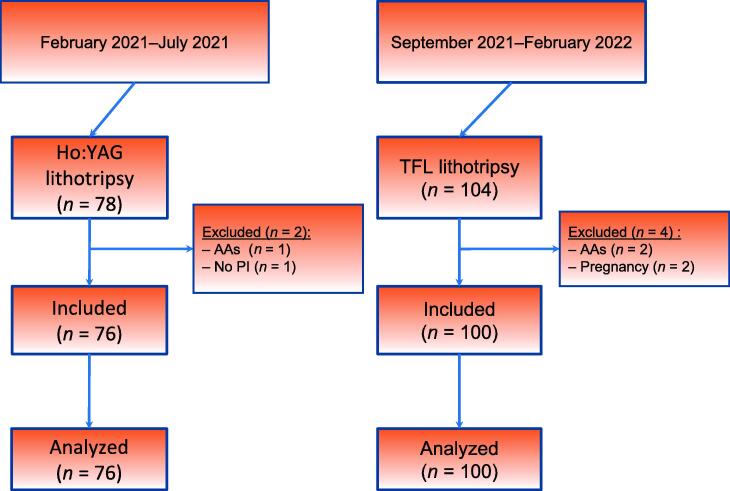
Study flow chart showing patient inclusion and exclusion according to the consecutive design. AAs = anatomic abnormalities; PI = preoperative imaging; TFL = thulium fiber laser.

**Fig. 2:**
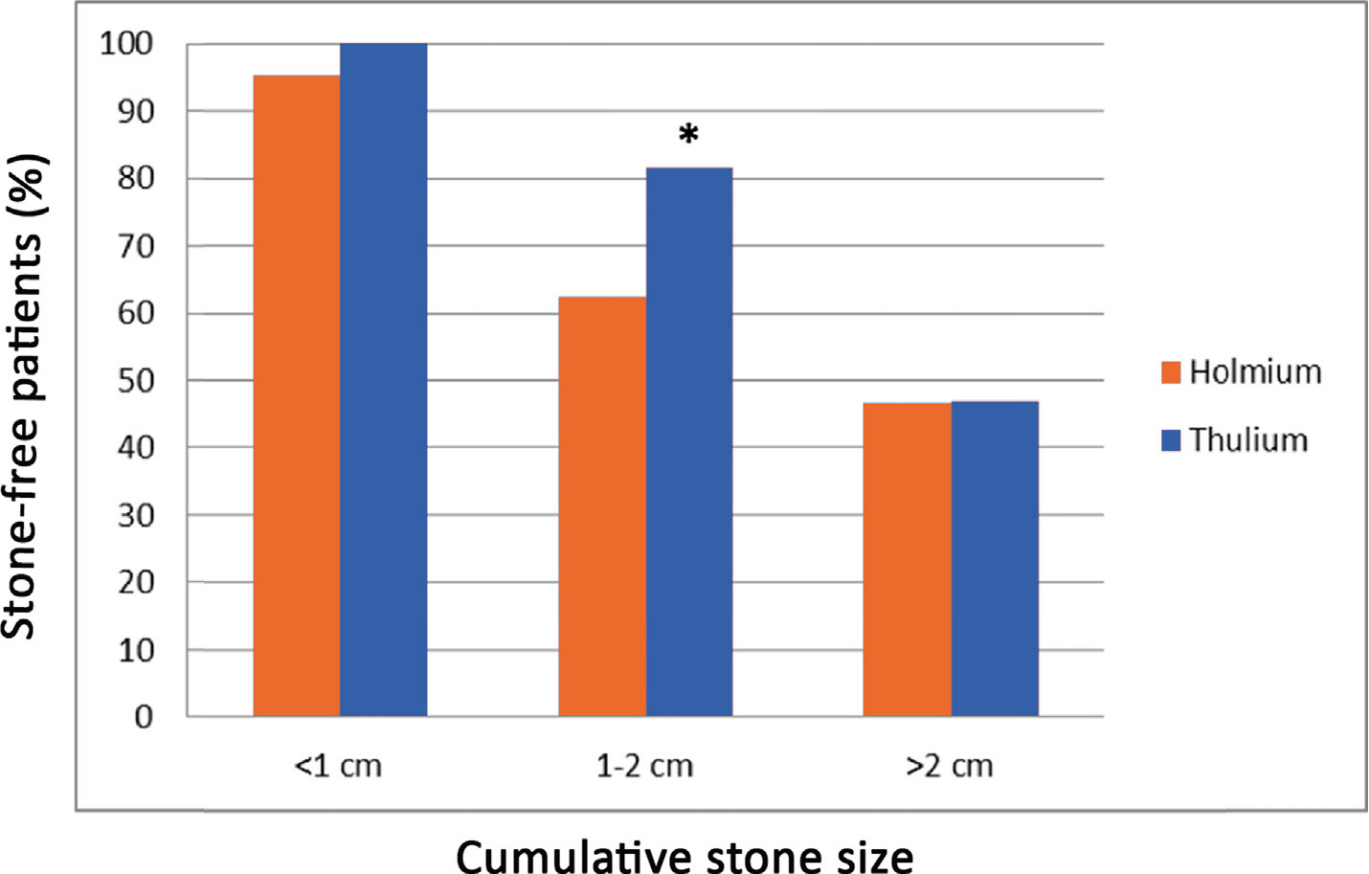
Proportion of stone-free patients by stone size and laser used. * *p* = 0.04.

**Fig. 3:**
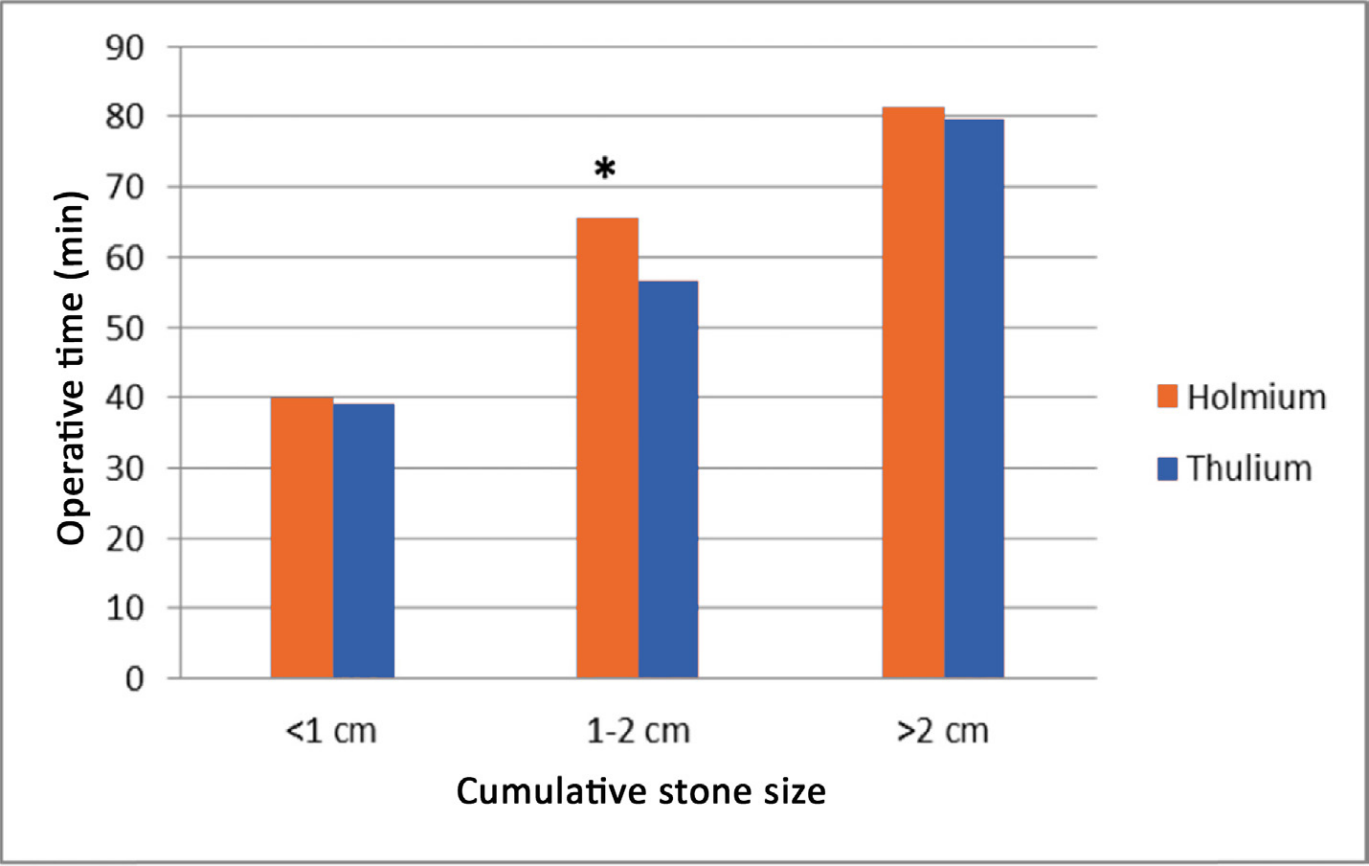
Operative time by stone size and laser used. * *p* = 0.04.

A total of 182 patients were treated during the study period, of whom 176 (76 Ho:YAG lithotripsy and 100 TFL) were included in the study (Fig. 1). Patient characteristics such as sex, age, body mass index, and stone composition were comparable between the groups. The mean cumulative stone size (20.4 vs 14.8 mm; *p* = 0.01) and mean diameter of the largest stone (14.6 vs 11.6 mm; *p* = 0.01) were significantly greater in the TFL than in the Ho:YAG group (Table 1). Stone size was not taken into consideration before the analysis because the study was designed to compare URS lithotripsy procedures during consecutive periods. The operative time and overall complication rate were similar in the two groups. Perioperative bleeding was less frequent in the TFL group (2% vs 11.8%; *p* = 0.001). Postoperative pain was similar in the two groups. In the TFL group, use of stone baskets was significantly less frequent (55% vs 90%; *p* < 0.001; Table 2). The SF rate was similar in the TFL and Ho:YAG groups (72% vs 68.4%; *p* = 0.06; Table 3). For a cumulative stone size of 1–2 cm, the SF rate at 3 mo was higher in the TFL than in the Ho:YAG group (81.6% vs 62.5%; *p* = 0.04; Fig.2) and the operative time was shorter in the TFL than in the Ho:YAG group (56.6 vs 65.6 min; *p* = 0.04; Fig. 3).

**Table 1:** Preoperative patient and stone characteristics

Parameter	Holmium (*n* = 76)	Thulium (*n* = 100)	*p* value
Male, *n* (%)	43 (56.6)	43 (43)	0.09
Mean age, yr (standard deviation)	57 (18.2)	60.1 (17.7)	0.26
Mean body mass index, kg/m^2^ (standard deviation)	27.6 (6.5)	27.2 (5)	0.63
Laterality, *n* (%)			0.40
Right kidney	39 (51.3)	40 (40)	
Left kidney	36 (47.4)	58 (58)	
Bilateral	1 (1.3)	1 (1)	
Kidney transplant	0 (0)	1 (1)	
Mean number of calculi, *n* (standard deviation)	1.6 (0.9)	1.9 (1.3)	0.11
Mean diameter of the largest stone, mm (standard deviation)	11.6 (5.6)	14.6 (8.8)	0.01
Mean cumulative stone size, mm (standard deviation)	14.8 (6.9)	20.4 (17.3)	0.01
Stone location, *n* (%)			
Kidney	61 (80.3)	88 (88)	0.21
Upper calyx	14 (23)	20 (22.7)	0.84
Middle calyx	19 (31.1)	23 (26.1)	0.85
Lower calyx	42 (68.9)	49 (55.7)	0.44
Renal pelvis	19 (31.1)	41 (46.6)	0.37
Ureter	20 (26.3)	23 (23)	0.72
Lumbar	13 (65)	15 (65.2)	0.83
Iliac	2 (10)	1 (4.3)	0.57
Pelvic	6 (30)	8 (34.8)	1.00
Anticoagulant therapy, *n* (%)	8 (10.5)	17 (17)	0.27
Neurological diseases, *n* (%)	5 (6.6)	13 (13)	0.21
Preoperative JJ stent, *n* (%)	56 (73.7)	61 (61)	0.85
Calculus type, *n* (%)			0.53
Calcium oxalate monohydrate	19 (25)	22 (22)	
Calcium oxalate dihydrate	1 (1.3)	5 (5)	
Uric acid	9 (11.8)	9 (9)	
Calcium phosphate	13 (17.1)	25 (25)	
Mixed	32 (42.1)	38 (38)

**Table 2:** Operative data

Parameter	Holmium (*n* = 76)	Thulium (*n* = 100)	*p* value
Surgeon expertise, *n* (%)			
Senior	15 (19.7)	32 (32)	0.09
Junior	61 (80.3)	68 (68)	
Mean operative time, min (standard deviation)	61.5 (25.8)	62 (25.5)	0.16
Access sheath, *n* (%)	65 (85.5)	75 (75)	0.09
Laser fiber size, *n* (%)			<0.001
150 μm	0 (0)	45 (45)	
200 μm	0 (0)	55 (55)	
270 μm	73 (96.1)	0 (0)	
375 μm	3 (3.9)	0 (0)	
Stone basket used, *n* (%)	69 (90.8)	55 (55)	<0.001
Ureteral catheter, *n* (%)	26 (34.2)	25 (25)	0.24
Terumo guidewire, *n* (%)	76 (100)	99 (99)	1.00
Sensor guidewire, *n* (%)	4 (5.2)	2 (2)	0.40
Bladder catheter, *n* (%)	10 (13.2)	5 (5)	0.06
Perioperative complications, *n* (%)	11 (14.5)	8 (8)	0.22
Bleeding	9 (81.8)	2 (25)	0.01
Broken ureteroscope	0 (0)	1 (13)	1.00
Urine leakage	3 (27.3)	5 (63)	1.00
Ureteral injury	1 (9.1)	1 (13)	1.00
Incomplete ureteroscopy, *n* (%)	9 (11.8)	12 (12)	1.00
Postoperative JJ stenting, *n* (%)	70 (92.1)	92 (92)	1.00

**Table 3:** Postoperative data

Parameter	Holmium (*n* = 76)	Thulium (*n* = 100)	*p* value
Mean postoperative pain score (standard deviation)	1.26 (1.7)	1.18 (1.6)	0.08
Ambulatory case, *n* (%)	64 (84.2)	82 (82)	0.84
Postoperative complications, *n* (%)	9 (11.8)	14 (14)	0.82
Clavien grade I–II	6 (66.7)	9 (64.3)	0.96
Clavien grade III–V	3 (33.3)	4 (28.6)	0.96
Pyelonephritis (%)	8 (10.5)	5 (5)	0.24
Urinoma, *n* (%)	1 (1.3)	0 (0)	0.43
Stone-free, *n* (%)	52 (68.4)	72 (72)	0.61

## 4 Discussion

In our study, SF status at 3 mo did not significantly differ between the Ho:YAG and TFL approaches for lithotripsy. However, stone size was not comparable between the groups, with greater cumulative stone size and diameter of the largest stone in the TFL group. Stone diameter measurement is reliable and remains the easiest tool for preoperative estimation of the operative time in clinical practice [13]. TFL lithotripsy seems to be more efficient, with a shorter operative time for stones of 1–2 cm in subgroup analysis. Ulvik et al [14] reported SF rates of 67% with Ho:YAG laser and 92% with TFL for URS lithotripsy of stones measuring 6–16 mm. Use of TFL may not impact the operative time for stones <1 cm because of the fixed period needed for insertion into the ureter or kidney, regardless of the fragmentation or dusting period. A retrospective review of 102 cases by Ryan et al [15] revealed a shorter average operation time in the TFL group compared to Ho:YAG, with 13 min saved per case (62.8 vs 49.8 min; *p* = 0.02). Many patients had a cumulative stone size >2 cm in our study. The American Urological Association, European Association of Urology, and CLAFU guidelines recommend offering percutaneous nephrolithotomy (PCNL) as first-line therapy for patients with a stone burden >2 cm [1], [11], [16]. PCNL is a difficult technique and is associated with higher mortality than that with URS. In some frail patients, several URS sessions may be used. The fragmentation rate of TFL means that it could be possible to consider extending the URS indications to larger stones. Moreover, use of TFL in mini-PCNL is safe and effective for stones measuring 10–20 mm, especially for lower calyceal stones [17].

No major complication due to the TFL was reported in our study. Our complication rate of 10–14% is comparable to the rates in recent studies with TFL [4], [5], [6], [7], [14], mostly involving pyelonephritis or acute renal colic pain and hematuria. We observed one case of pyelo-ureteral stenosis in each group, despite the total energy required and the rise in temperature in the urinary tract during laser lithotripsy described for Ho:YAG and TFL [18], [19]. The higher cost of the TFL device can be balanced against the lower use of stone baskets in our study. Endoscopic identification of stone composition, with surface and cross-sectional photos during endoscopy, may be useful in avoiding the need to basket fragments for spectrophotometry [20]. In addition, stone dust samples can be drawn through the ureteroscope or the access sheath and sent for morphocompositional analysis [21], [22].

Our study has some limitations, such as the small group size and the single-center design. Owing to the lack of randomization, the two initial groups differed in stone size. However, we took this factor into account during statistical analysis, stratifying our cohort according to different stone-size cutoffs. Furthermore, stone volume is more reliable than stone size in comparing operative time [23]. This study was designed at the beginning of TFL use and the laser settings were decided by each surgeon, so it is likely that there was significant heterogeneity. There are actually no official recommendations regarding optimal settings, which remain operator-dependent [24]. We included many patients with a stone burden >2 cm. The SF status may be underestimated, because at least two procedures are needed in such cases.

## 5 Conclusions

TFL is an effective and safe laser for URS treatment of UUT lithiasis. SF status at 3 mo was comparable between the TFL and Ho:YAG lasers in our study, with a low rate of complications. Further randomized studies are necessary to confirm the TFL performance in lithotripsy in relation to stone size.

  ***Author contributions***: Bertrand Delbarre had full access to all the data in the study and takes responsibility for the integrity of the data and the accuracy of the data analysis.

  *Study concept and design*: All authors.

*Acquisition of data*: Delbarre, Ferragu.

*Analysis and interpretation of data*: Delbarre, Bigot.

*Drafting of the manuscript*: Delbarre, Bigot.

*Critical revision of the manuscript for important intellectual content*: Culty, Lebdai, Bigot.

*Statistical analysis*: Bigot.

*Obtaining funding*: None.

*Administrative, technical, or material support*: Delbarre.

*Supervision*: Bigot.

*Other*: None.

  ***Financial disclosures:*** Bertrand Delbarre certifies that all conflicts of interest, including specific financial interests and relationships and affiliations relevant to the subject matter or materials discussed in the manuscript (eg, employment/affiliation, grants or funding, consultancies, honoraria, stock ownership or options, expert testimony, royalties, or patents filed, received, or pending), are the following: None.

  ***Funding/Support and role of the sponsor*:** None.

## References

[1] Quhal F., Seitz C. (2021). Guideline of the guidelines: urolithiasis. Curr Opin Urol.

[2] Panthier F., Doizi S., Corrales M., Traxer O. (2021). Pulsed lasers and endocorporeal laser lithotripsy. Prog Urol.

[3] Panthier F., Doizi S., Lapouge P. (2021). Comparison of the ablation rates, fissures and fragments produced with 150 μm and 272 μm laser fibers with superpulsed thulium fiber laser: an in vitro study. World J Urol.

[4] Carrera R.V., Randall J.H., Garcia-Gil M. (2021). Ureteroscopic performance of high power super pulse thulium fiber laser for the treatment of urolithiasis: results of the first case series in North America. Urology.

[5] Corrales M., Traxer O. (2021). Initial clinical experience with the new thulium fiber laser: first 50 cases. World J Urol.

[6] Enikeev D., Taratkin M., Klimov R. (2020). Superpulsed thulium fiber laser for stone dusting: in search of a perfect ablation regimen—a prospective single-center study. J Endourol.

[7] Sierra A., Corrales M., Kolvatzis M., Traxer O. (2022). Initial clinical experience with the thulium fiber laser from Quanta System: first 50 reported cases. World J Urol.

[8] Kronenberg P., Hameed B.Z., Somani B. (2021). Outcomes of thulium fibre laser for treatment of urinary tract stones: results of a systematic review. Curr Opin Urol.

[9] Quirke K., Aydin A., Brunckhorst O. (2018). Learning curves in urolithiasis surgery: a systematic review. J Endourol.

[10] Clavien P.A., Barkun J., de Oliveira M.L. (2009). The Clavien-Dindo classification of surgical complications: five-year experience. Ann Surg.

[11] Chabannes É., Bensalah K., Carpentier X. (2013). [Management of adult’s renal and ureteral stones. Update of the Lithiasis Committee of the French Association of Urology (CLAFU). General considerations]. Prog Urol.

[12] Ghani K.R., Wolf J.S., Wolf J.S. (2015). What is the stone-free rate following flexible ureteroscopy for kidney stones?. Nat Rev Urol.

[13] Diamand R., Idrissi-Kaitouni M., Coppens E., Roumeguère T., Legrand F. (2018). [Evaluation of stone size before flexible ureteroscopy: which measurement is best?]. Prog Urol.

[14] Ulvik Ø., Sørstrand Æsøy M., Juliebø-Jones P., Gjengstø P., Beisland C. (2022). Thulium fibre laser versus holmium:YAG for ureteroscopic lithotripsy: outcomes from a prospective randomised clinical trial. Eur Urol.

[15] Ryan J.R., Nguyen M.H., Linscott J.A. (2022). Ureteroscopy with thulium fiber laser lithotripsy results in shorter operating times and large cost savings. World J Urol.

[16] Assimos D., Krambeck A., Miller N.L. (2016). Surgical management of stones: American Urological Association/Endourological Society guideline, part I. J Urol.

[17] Perri D., Berti L., Pacchetti A. (2022). A comparison among RIRS and MiniPerc for renal stones between 10 and 20 mm using thulium fiber laser (Fiber Dust): a randomized controlled trial. World J Urol.

[18] Jiang Y.L., Peng C.X., Wang H.Z., Qian L.J. (2019). Comparison of the long-term follow-up and perioperative outcomes of partial nephrectomy and radical nephrectomy for 4 cm to 7 cm renal cell carcinoma: a systematic review and meta-analysis. BMC Urol.

[19] Kraft L., Petzold R., Suarez-Ibarrola R., Miernik A. (2022). In vitro fragmentation performance of a novel, pulsed thulium solid-state laser compared to a thulium fibre laser and standard Ho:YAG laser. Lasers Med Sci.

[20] Bergot C., Robert G., Bernhard J.C. (2019). [The basis of endoscopic stones recognition, a prospective monocentric study.]. Prog Urol.

[21] Keller E.X., De Coninck V., Doizi S., Daudon M., Traxer O. (2021). Thulium fiber laser: ready to dust all urinary stone composition types?. World J Urol.

[22] Sierra A., Corrales M., Kolvatzis M., Daudon M., Traxer O. (2022). Thulium fiber laser’s dust for stone composition analysis: is it enough? A pilot study. J Endourol.

[23] Sorokin I., Cardona-Grau D.K., Rehfuss A. (2016). Stone volume is best predictor of operative time required in retrograde intrarenal surgery for renal calculi: implications for surgical planning and quality improvement. Urolithiasis.

[24] Sierra A., Corrales M., Piñero A., Traxer O. (2022). Thulium fiber laser pre-settings during ureterorenoscopy: Twitter’s experts’ recommendations. World J Urol.

